# Psychological distress and health-related quality of life up to 2 years after oesophageal cancer surgery: nationwide population-based study

**DOI:** 10.1093/bjsopen/zraa038

**Published:** 2021-01-09

**Authors:** Y J Liu, A Schandl, S Markar, A Johar, P Lagergren

**Affiliations:** Surgical Care Science, Department of Molecular Medicine and Surgery, Karolinska Institutet, Karolinska University Hospital, Stockholm, Sweden; Surgical Care Science, Department of Molecular Medicine and Surgery, Karolinska Institutet, Karolinska University Hospital, Stockholm, Sweden; Department of Anaesthesiology and Intensive Care, Södersjukhuset, Stockholm, Sweden; Surgical Care Science, Department of Molecular Medicine and Surgery, Karolinska Institutet, Karolinska University Hospital, Stockholm, Sweden; Department of Surgery and Cancer, Imperial College London, London, UK; Surgical Care Science, Department of Molecular Medicine and Surgery, Karolinska Institutet, Karolinska University Hospital, Stockholm, Sweden; Surgical Care Science, Department of Molecular Medicine and Surgery, Karolinska Institutet, Karolinska University Hospital, Stockholm, Sweden; Department of Surgery and Cancer, Imperial College London, London, UK

## Abstract

**Background:**

Patients are at higher risk of suffering from psychological distress and reduced health-related quality of life (HRQoL) after oesophageal cancer surgery. This Swedish nationwide population-based longitudinal study aimed to evaluate the association between psychological distress and HRQoL up to 2 years after oesophageal cancer surgery.

**Methods:**

The study included patients with oesophageal cancer who had survived for 1 year after oesophageal cancer surgery. The exposure was psychological distress measured using the Hospital Anxiety and Depression Scale. Patients scoring at least 8 on either the anxiety or the depression subscale were classified as having psychological distress. The outcome was HRQoL assessed by the European Organisation for Research and Treatment of Cancer Quality of Life Questionnaire generic and disease-specific questionnaires (EORTC QLQ-C30 and QLQ-OG25). Exposure and outcome were measured at 1, 1.5, and 2 years after operation. Fixed-effects models with adjustment for all time-invariant confounding and potential time-varying confounders were used to examine the mean score difference in HRQoL between patients with and without psychological distress.

**Results:**

In total, 180 patients were analysed. Clinically relevant, statistically significant and time-constant mean score differences were found in emotional function, social function, dyspnoea, anxiety, eating difficulty, eating in front of others, and weight loss (mean score difference range 10–29). Mean score differences for global quality of life, cognitive function, appetite loss, EORTC QLQ-C30 summary score, and trouble with taste increased over time, and reached clinical and statistical significance at 1.5 and/or 2 years after surgery. For body image, there was a clinically relevant decrease in mean score difference over time.

**Conclusion:**

Psychological distress was associated with several aspects of poor HRQoL up to 2 years after surgery for oesophageal cancer.

## Teaser

Patients are at higher risk of suffering from psychological distress and reduced health-related quality of life (HRQoL) after oesophageal cancer surgery. This nationwide population-based longitudinal study demonstrated that psychological distress was associated with several aspects of poor HRQoL for up to 2 years after oesophageal cancer surgery.

## Resumen

**Antecedentes:**

Los pacientes con cáncer de esófago presentan un riesgo mayor de sufrir problemas psicológicos y tener una menor calidad de vida relacionada con la salud (*health-related quality of life*, HRQL) tras la cirugía. Este estudio longitudinal sueco de base poblacional y nacional se propuso evaluar la asociación entre problemas psicológicos y HRQL hasta dos años después de la cirugía del cáncer de esófago.

**Métodos:**

En total, se analizaron 180 pacientes con cáncer de esófago que habían sobrevivido un año tras la cirugía. La exposición fue el problema psicológico medido con la *Hospital Anxiety and Depression Scale*. Los pacientes con una puntuación de ≥ 8 ya fuere en la subescala de ansiedad o de depresión se clasificaron como portadores de un problema psicológico. El resultado fue la HRQL evaluada con los cuestionarios genéricos y específicos de la *European Organisation for Research and Treatment of Cancer Qu*ality (EORTC QLQ-C30 y QLQ-OG25). La exposición y el resultado fueron medidos al cabo de 1, 1,5 y 2 años tras la operación. Se utilizaron modelos de efectos fijos con ajustes para todos los factores de confusión, tanto variables como invariables con el tiempo, a fin de analizar la diferencia de la puntuación media de HRQL entre pacientes con y sin problemas psicológicos.

**Resultados:**

Las diferencias de las puntuaciones medias de la función emocional, función social, disnea, ansiedad, dificultad para comer, comer con otros y pérdida de peso fueron clínicamente relevantes y estadísticamente significativas (rango de la diferencia de la puntuación media 10 a 29). En la calidad de vida global, la función cognitiva, la pérdida de apetito, la puntuación global del EORTC QLQ-C30 y los problemas con el gusto, las diferencias en la puntuación media aumentaron con el tiempo y alcanzaron significación clínica y estadística al cabo de 1,5 y/o 2 años tras la cirugía, mientras que en la imagen corporal, la diferencia clínicamente relevante de la puntuación media disminuyó con el tiempo.

**Conclusión:**

Los problemas psicológicos se asociaron con una HRQL pobre en varios de los dominios de calidad de vida en pacientes con cáncer de esófago tras la cirugía.

## Introduction

Oesophageal cancer is the seventh most common cancer and the sixth leading cause of cancer death globally^1^. The main treatment with curative intent, surgical resection, is an extensive procedure with substantial detrimental effects on patients’ health-related quality of life (HRQoL)^2^^,^^3^. In addition, patients with oesophageal cancer, especially those who are surgically treated, are at higher risk of suffering from psychological distress than the general population^4^^,^^5^. Previous studies^6–12^ have demonstrated that psychological distress is related to impaired HRQoL among patients with other subtypes of cancer. For patients with oesophageal cancer, one study^13^ reported an association between psychological distress and poor HRQoL before oesophagectomy, and at 1 and 3 months afterwards. Whether this association continues to exist more than 3 months after surgery remains unknown.

Although previous studies have performed multiple regression analyses, and found an association between psychological distress and HRQoL, none adjusted for unobserved variables. Unobserved factors such as personality traits could be the common cause of psychological distress and poor HRQoL^14–18^, which might mean that the reported association between psychological distress and HRQoL has been overestimated.

Identifying the correct association between psychological distress and HRQoL among patients who have undergone oesophageal cancer surgery is important. It may not only help patients and healthcare providers build a proper expectation about postoperative life changes, but might also contribute to the early detection of psychological distress and poor HRQoL, leading to timely supportive interventions. Given that both psychological distress and decreased HRQoL are predictors of poor survival after oesophageal cancer surgery^4^^,^^19^^,^^20^, the amelioration of psychological distress and improvement of HRQoL may contribute to survival.

The aim of this study was to use Swedish nationwide population-based longitudinal data to assess the association between psychological distress and HRQoL, with adjustment for both observed time-varying confounders and all time-invariant factors.

## Methods

###  

A prospective, ongoing Swedish nationwide population-based cohort study entitled OSCAR (Oesophageal Surgery on Cancer patients—Adaptation and Recovery) was launched on 1 January 2013. It follows up survivors after curatively intended oesophageal cancer surgery and their closest family members from 1 to 5 years after surgery. A detailed description of the OSCAR study has been published elsewhere^21^. In brief, a project coordinator contacts the pathology departments at eight hospitals performing oesophagectomy in Sweden to identify potential participants. All patients who have survived for 1 year after oesophagectomy for cancer are invited to participate. A research nurse visits the patient for the 1- and 5-year interviews, whereas follow-up at other time points is conducted by mailing paper questionnaires. Clinical data are obtained from medical records, the Swedish Patient Registry, and the Swedish Cancer Registry. Patient demographics are retrieved from the Swedish Longitudinal Integration Database for Health Insurance and Labour Market Studies, and the national health data registries. Information about mortality is collected through linkage to the Swedish Register of the Total Population and the Swedish Cause of Death register. The study was approved by the Regional Ethical Review Board in Stockholm, Sweden (diary number 2013/844-31/1) and informed consent was obtained from all participants before inclusion.

### Study participants

**Fig. 1:**
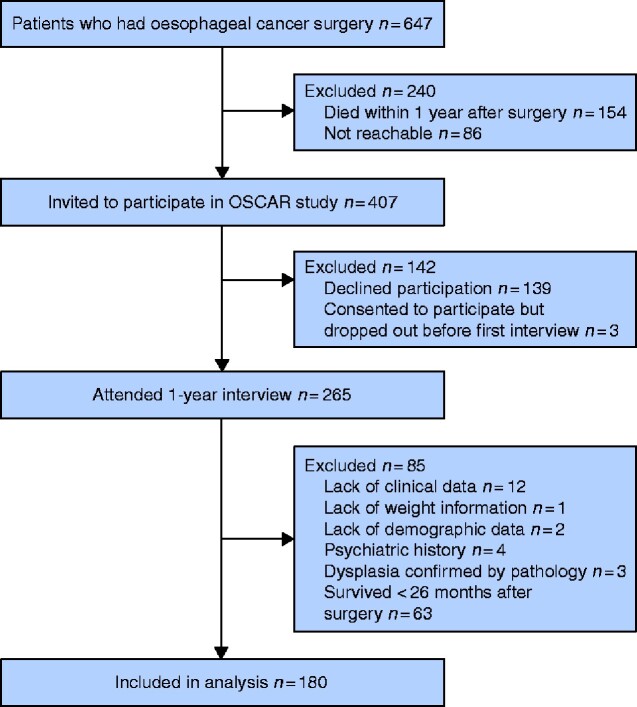
Study flow chart

The present longitudinal study was based on the most recent data from the OSCAR study, and included patients undergoing oesophageal cancer surgery between 1 January 2013 and 28 February 2018 in Sweden. Three follow-up time points, 1, 1.5, and 2 years after surgery were included. The process of patient selection is outlined in*Fig. 1*. To account for the possible concern that patient-reported outcomes might be affected by cancer recurrence and likelihood of death, patients who survived less than 26 months were excluded along with those who lacked clinical or sociodemographic information, and those with a history of psychiatric disorders or resection for premalignant (dysplasia) lesions.

### Exposure: psychological distress

Psychological distress was assessed at 1, 1.5, and 2 years after surgery using the Hospital Anxiety and Depression Scale (HADS)^22^^,^^23^. This is a well-validated and widely used instrument with two subscales measuring anxiety and depression separately. Each subscale has seven questions and each question is graded on a four-point Likert scale ranging from 0 to 3, where a higher score represents a greater burden of anxiety or depression. A score of at least 8 on each subscale indicates a possible–probable case of anxiety or depression^22^^,^^23^.

The exposure group included patients who scored at least 8 on either the anxiety or depression subscale. The unexposed group, comprising patients without psychological distress, included those who scored less than 8 on both the anxiety and depression subscales.

### Outcome: HRQoL

HRQoL was measured at 1, 1.5, and 2 years after surgery using the European Organisation for Research and Treatment of Cancer Quality of Life Questionnaire – Core 30 (EORTC QLQ-C30) and disease site-specific (Oesophago-Gastric) module (EORTC QLQ-OG25)^24^^,^^25^. Both questionnaires have shown good psychometric properties^24^^,^^25^.

The EORTC QLQ-C30 is a multidimensional measure with 30 items measuring HRQoL aspects related to patients with cancer in general. The questionnaire includes one global quality-of-life scale, five functional scales (physical, role, emotional, social, cognitive), three symptom scales (fatigue, pain, nausea/vomiting), and six single items (dyspnoea, appetite loss, insomnia, constipation, diarrhoea, financial difficulty). All items are scored using a four-point Likert scale from 1 (not at all) to 4 (very much), except for the global quality-of-life scale which ranges from 1 (very poor) to 7 (excellent)^24^.

The EORTC QLQ-OG25 is a 25-item module comprising six symptom scales (dysphagia, eating difficulty, reflux, odynophagia, pain and discomfort, anxiety) and ten single items (eating in front of others, dry mouth, trouble with taste, body image, trouble swallowing saliva, choking when swallowing, trouble with coughing, trouble talking, weight loss, hair loss)^25^. Each item is scored on a four-point Likert scale as for the EORTC QLQ-C30.

The raw score for each HRQoL scale was linearly transformed to a 0–100 scale according to the EORTC Scoring Manual, with a higher score representing better function/global quality of life or higher symptom burden^26^. A single summary score for the EORTC QLQ-C30 was calculated according to the guideline^27^.

### Statistical analysis

Characteristics of patients with and without psychological distress were summarized and compared at each time point using Fisher’s exact test. There were very few missing data in HADS and HRQoL questionnaires, and these were handled with mean imputation^26^^,^^28^.

To take the non-independence of the repeated measures into account, a random-effects model was used to assess the mean score differences for each HRQoL aspect between patients with and without psychological distress. Adjustments were made for time, age, sex, cohabitation status, educational level, Charlson Co-morbidity Index^29^, tumour stage, histology, postoperative complication within 30 days after surgery, and weight change after oesophagectomy. Psychological distress and weight change were treated as time-varying variables, and the interaction between psychological distress and time was examined using the Wald test.

Because a random-effects model can control only for observed confounders, a fixed-effects model was also used to adjust for potential unmeasured time-invariant confounding^30^. Compared with the random-effects model, a fixed-effects model allows unobserved variables to be associated with all observed variables and uses only within-individual differences^30^. Therefore, only time-varying independent variables, including psychological distress, weight change, and assessment time points, needed to be included in the model.

The outcome HRQoL has many aspects; to minimize the type I error due to multiple comparisons, the statistical significance of adjusted mean score differences was examined only if they had clinical relevance. Based on the evidence-based interpretation guidelines, the adjusted mean score differences were classified as: trivial (circumstances unlikely to have any clinical relevance or where there was no difference), small (subtle but clinically relevant), medium (likely to be clinically relevant but to a lesser extent) or large (of unequivocal clinical relevance)^31^^,^^32^. Only medium and large differences were regarded as clinically relevant in the present study. For aspects where no such defined cut-offs were recommended, adjusted mean score differences of between 10 and 20 were considered to be of medium clinical relevance, whereas adjusted mean score differences greater than 20 were considered to be of large clinical relevance^33^^,^^34^.

All statistical analyses were performed using Stata^®^ version 13 (StataCorp, College Station, Texas, USA) and SAS^®^ version 9.4 (SAS Institute, North Carolina, USA). All 95 per cent confidence intervals were two-sided.

## Results

In the specified time interval, 407 patients were invited to take part in the OSCAR study, of whom 265 (65.1 per cent) consented and attended the 1-year interview (*Fig. 1*). Of these, 85 patients were excluded, leaving 180 patients in the analysis. Follow-up data were available for 157 patients at 1.5 years and for 151 patients at 2 years after surgery.

Characteristics of the included 180 patients are shown in *Table 1*. The mean(s.d.) age was 66.4(8.5) (range 38.2–83.7) years and 85.6 per cent of the patients were men. At 1 year after surgery, 19 patients (10.6 per cent) reported psychological distress. The proportion increased to 27 of 157 (17.2 per cent) at 1.5 years and 35 of 151 (23.2 per cent) at 2 years. Characteristics of patients with and without psychological distress at the three assessment time points are summarized in *Table S1*. No statistically significant difference was found between patients with and without psychological distress at the three assessment time points. Patients who did not answer the 2-year follow-up questionnaires seemed to have higher tumour stage and more severe postoperative complications (*Table S2*).

**Table 1:** Characteristics of included patients who had surgery for oesophageal cancer

	**No. of patients** **(*n* = 180)**
**Age at surgery (years)**	
Mean (standard deviation)	66.4 (8.5)
Range	38.2–83.7
< 60	40 (22.2)
60–74	111 (61.7)
≥ 75	29 (16.1)
**Sex ratio (F : M)**	26 : 154
**Cohabitation status**	
Non-cohabitating	42 (23.3)
Cohabitating	138 (76.7)
**Education level**	
Nine-year compulsory school	44 (24.4)
Upper secondary school	81 (45.0)
Higher education	55 (30.6)
**Charlson Co-morbidity Index score**	
0	87 (48.3)
1	55 (30.6)
≥ 2	38 (21.1)
**Neoadjuvant therapy**	
No	33 (18.3)
Yes	147 (81.7)
**Tumour histology**	
Adenocarcinoma	151 (83.9)
Squamous cell carcinoma	29 (16.1)
**Surgical approach**	
Total minimally invasive oesophagectomy	50 (27.8)
Hybrid minimally invasive oesophagectomy	59 (32.8)
Open oesophagectomy	71 (39.4)
**Tumour stage**	
I	67 (37.2)
II	59 (32.8)
III–IV	54 (30.0)
**Postoperative complications (Clavien–Dindo grade)**	
None	65 (36.1)
I–II	50 (27.8)
III–IV	65 (36.1)

Values in parentheses are percentages unless indicated otherwise.

### HRQoL differences between patients with and without psychological distress

After adjustment for observed potential confounders (random-effects model) between patients with and without psychological distress, clinically relevant and statistically significant mean score differences were found in almost all aspects of HRQoL, except physical function, role function, constipation, dysphagia, reflux, pain and discomfort, trouble with coughing, and trouble talking (*Tables*  *S3* and *S4*). After controlling for all time-invariant variables, via fixed-effects models, the adjusted mean score differences between patients with and without psychological distress were attenuated in all aspects, and became clinically irrelevant in some areas including fatigue, nausea/vomiting, pain, insomnia, diarrhoea, financial difficulty, odynophagia, dry mouth, trouble swallowing saliva, and choking when swallowing (*Tables 2* and *3*, *Tables*  *S3* and *S4*).

**Table 2:** Adjusted mean score difference in aspects of health-related quality of life between patients with and without psychological distress at 1, 1.5, and 2 years after surgery for oesophageal cancer: results from fixed-effects models without significant time interaction

	**Adjusted mean score difference**
**EORTC QLQ-C30**	
Emotional function	–19 (–25, –14)*
Social function	–17 (–24, –9)*
Dyspnoea	11 (2, 20)*
Physical function	–8 (–12, –4)
Role function	–11 (–18, –4)
Fatigue	12 (6, 18)
Nausea/vomiting	2 (–4, 8)
Pain	12 (5, 18)
Insomnia	11 (1, 20)
Diarrhoea	5 (–3, 14)
Financial difficulty	8 (2, 13)
Constipation	6 (–1, 14)
**EORTC QLQ-OG25**	
Anxiety	29 (21, 36)*
Eating difficulty	13 (7, 18)*
Eating in front of others	10 (2, 17)*
Weight loss	13 (5, 21)*
Odynophagia	7 (1, 13)
Dry mouth	8 (+0, 16)
Trouble swallowing saliva	7 (2, 13)
Choking when swallowing	8 (1, 15)
Dysphagia	5 (1, 9)
Reflux	–2 (–9, 6)
Pain and discomfort	0 (–6, 7)
Trouble with coughing	5 (–3, 13)
Trouble talking	9 (3, 14)

Values are mean differences, with 95 per cent confidence intervals in parentheses, rounded to the nearest integer. EORTC, European Organisation for Research and Treatment of Cancer; QLQ-C30, Quality of Life Questionnaire – Core 30; QLQ-OG25, Quality of Life Questionnaire – Oesophago-Gastric module 25. *Clinically relevant and statistically significant.

**Table 3:** Adjusted mean score difference in aspects of health-related quality of life between patients with and without psychological distress at 1, 1.5, and 2 years after surgery for oesophageal cancer: results from fixed-effects models with significant time interaction

	Adjusted mean score difference		
	1 year	1.5 years	2 years
**EORTC QLQ-C30**			
Global quality of life	–5 (–15, 4)	–13 (–22, 5)*	–20 (–28, –13)*
Cognitive function	–7 (–14, –0)	–3 (–9, 3)	–11 (–17, –6)*
Appetite loss	6 (–6, 18)	7 (–3, 17)	26 (17, 35)*
Summary score	–8 (–13, –3)	–8 (–12, –4)	–14 (–18, –10)*
**EORTC QLQ-OG25**			
Trouble with taste	–4 (–15, 7)	13 (3, 23)*	5 (–4, 13)
Body image	28 (17, 38)*	23 (14, 32)*	13 (5, 21)*

Values are mean differences, with 95 per cent confidence intervals in parentheses, rounded to the nearest integer. EORTC, European Organisation for Research and Treatment of Cancer; QLQ-C30, Quality of Life Questionnaire – Core 30; QLQ-OG25, Quality of Life Questionnaire – Oesophago-Gastric module 25.

*Clinically relevant and statistically significant.

Compared with patients without psychological distress, those with psychological distress reported clinically relevant and statistically significantly worse emotional function (mean score difference –19, 95 per cent c.i. –25 to –14) and social function (mean score difference –17, 95 per cent c.i. –24 to –9), and more problems with dyspnoea (mean score difference 11, 95 per cent c.i. 2 to 20), anxiety (mean score difference 29, 95 per cent c.i. 21 to 36), eating difficulty (mean score difference 13, 95 per cent c.i. 7 to 18), eating in front of others (mean score difference 10, 95 per cent c.i. 2 to 17), and worry regarding weight loss (mean score difference 13, 95 per cent c.i. 5 to 21) over the three assessment time points (*Table 2*). In addition, clinically relevant but time-varying mean score differences were found in global quality of life, cognitive function, appetite loss, EORTC QLQ-C30 summary score, trouble with taste, and body image (*Table 3*).

### Effect modification by time and longitudinal changes in HRQoL

**Fig. 2:**
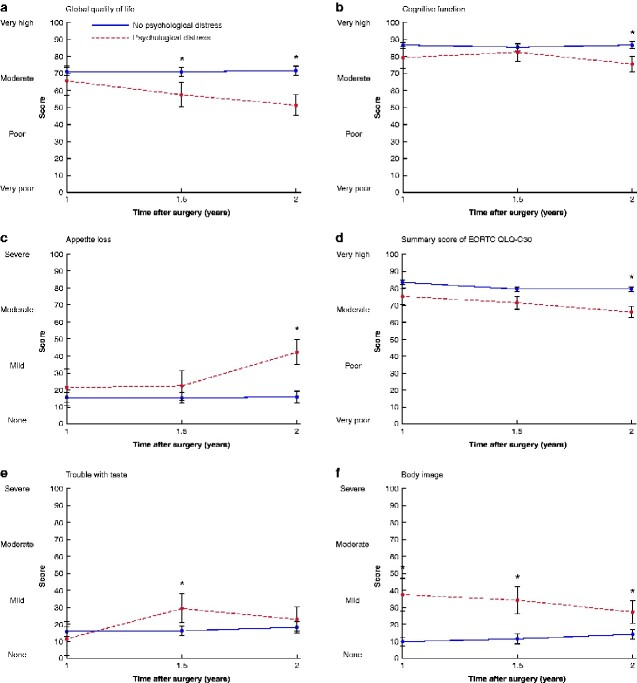
Estimated marginal means with 95 per cent confidence intervals of health-related quality of life scores at 1, 1.5, and 2 years after surgery for oesophageal cancer in patients with and without psychological distress **a** Global quality of life, **b** cognitive function, **c** appetite loss, **d** European Organisation for Research and Treatment of Cancer Quality of Life Questionnaire – Core 30 (EORTC QLQ-C30) summary score, **e** trouble with taste, and **f** body image. The results are from fixed-effects models. *Clinically relevant mean score difference in health-related quality of life between patients with and without psychological distress.

For global quality of life, cognitive function, appetite loss, EORTC QLQ-C30 summary score, and trouble with taste, the adjusted mean score differences increased over time, and reached clinical relevance and statistical significance at 1.5 and/or 2 years after surgery (*Table 3*). In these aspects, patients with psychological distress reported worse HRQoL over time, whereas patients without psychological distress reported relatively stable HRQoL scores at each time point (*Figs 2a–e*, *Table S5*).

In contrast, the adjusted mean score difference for body image decreased over time, even though it remained clinically relevant and statistically significant over the three assessment time points (*Table 3*). Patients without psychological distress reported similar self-perception of body image at each time point, whereas those with psychological distress reported relatively better body image (reduced self-doubt about body image) over time (*Fig. 2f*, *Table S5*).

## Discussion

This nationwide population-based longitudinal study indicated that psychological distress was associated with poor HRQoL up to 2 years after oesophageal cancer surgery in the aspects global quality of life, cognitive function, emotional function, social function, dyspnoea, appetite loss, EORTC QLQ-C30 summary score, anxiety, eating difficulty, eating in front of others, worry regarding weight loss, trouble with taste, and body image.

One single-centre study^13^ found that psychological distress and HRQoL were correlated before oesophagectomy and up to 3 months after surgery. The present analysis demonstrated that this association continued for up to 2 years after surgery. No previous study has examined the association between psychological distress and HRQoL with adjustment for unobserved time-invariant variables such as personality traits^6–13^, which can affect both psychological status and HRQoL^14–18^, and potentially lead to an overestimated or spurious association between these two variables. The present study eliminated such potential confounding bias by use of a longitudinal design and fixed-effects models. The use of evidence-based guidelines to evaluate the clinical relevance of an adjusted mean score difference before assessing its statistical significance minimized the risk of chance findings.

The study also has limitations. Because psychological distress and HRQoL were measured at the same time point, it was hard to determine the direction of potential causality between these two variables. There might be a reciprocal or bidirectional effect between them but, owing to the limited sample size, it was not feasible to include more time points and examine this. Although a fixed-effects model can control for all time-invariant confounding, it cannot adjust for unobserved time-varying variables such as cancer recurrence or time-varying treatments^30^. The present study lacked information on cancer recurrence; to account for the possible influence of cancer recurrence and the associated likelihood of death on patient-reported outcomes, only patients who had survived at least 2 more months after the last follow-up, at 2 years after surgery, were included. It is accepted that the effects of cancer recurrence and associated treatment might still exist, and the observed effect modification by time found in this study might result from such time-varying factors. In addition, patients with psychological distress and poor HRQoL might be more likely to decline participation and ignore the follow-up questionnaires, which means that the observed associations may have been underestimated. Finally, although HADS has good psychometric properties and has been recommended for use as a screening tool in oncological settings^35^, it might underestimate the psychological distress owing to insufficient coverage of assessed symptoms^36^. The cut-off point of 8 used to determine the possible–probable case of anxiety and depression, and the classification of patients as having psychological distress if they scored 8 or more on either subscale, should have reduced the likelihood of underestimation to some extent.

In almost all HRQoL aspects, the estimated mean score differences in the fixed-effects models were smaller than those obtained by the random-effects models. This attenuation suggests the existence of unobserved confounders that cause both psychological distress and poor HRQoL. Identifying these unobserved factors, especially the modifiable ones, may help predict and prevent psychological distress and improve HRQoL.

After further controlling for all time-invariant factors, clinically relevant, statistically significant, and time-constant mean score differences were found only in emotional function, social function, dyspnoea, anxiety, eating difficulty, eating in front of others, and worry regarding weight loss. It was expected that patients with psychological distress would report worse emotional function and more anxiety, but the exact mechanisms for the associations found in other aspects remain unclear. Because the adjusted mean score differences were time-constant, it is less likely that these associations were biased by time-varying confounders such as cancer recurrence. One possible explanation is that psychological distress might drive patients to behave differently^37^, thus leading to poor HRQoL. For example, patients with depression might tend to have social withdrawal and feel worthless^37^, which might lead to reduced social function and more trouble in eating in front of others. Anxiety might make patients worry more about their weight regardless of actual weight change. A previous study^38^ showed that psychological distress is an important determinant for development of dyspnoea. These observed associations highlight the importance of early detection of psychological distress and timely interventions that might improve patients’ HRQoL. Eating is a challenge for most patients after oesophagectomy^39^, and those who fail to overcome this difficulty might be more likely to develop psychological distress. This underlines the importance of timely dietary interventions to relieve eating difficulty, which might help prevent psychological distress and improve overall HRQoL^40^.

The present study also found that adjusted mean score differences increased from clinical irrelevance to relevance over time in global quality of life, cognitive function, appetite loss, EORTC QLQ-C30 summary score, and trouble with taste. The mechanisms for these effect modifications with time are unclear. Patients without psychological distress reported similar HRQoL scores at each time point, whereas those with psychological distress reported worse HRQoL over time. Given that some patients with psychological distress at later time points also reported psychological distress at preceding time points, one possible explanation is that HRQoL deteriorated further in patients with recurrent psychological distress, supporting the call for timely psychological screening and interventions. Given that appetite loss and trouble with taste are related to cancer recurrence, there is a possibility that the observed time-varying associations in these two aspects were spurious and caused by the time-varying common cause, cancer recurrence. In addition, the adjusted mean score difference in body image became smaller over time. This might be because patients adapted gradually to the changes after oesophagectomy with time^41^.

Psychological distress is associated with poor HRQoL in several aspects after oesophagectomy. For those affected, the effect is long-lasting. Although the causal direction of the association remains to be clarified, this study highlights the need for early psychological screening and timely interventions to ameliorate psychological distress and improve HRQoL in order to influence oesophageal cancer survivorship^4^^,^^19^^,^^20^.

## Funding

Swedish Cancer Society

Stockholm County Council (ALF Project)

Cancer Research Funds of Radiumhemmet

Swedish Research Council

Karolinska Institute and China Scholarship Council joint programme

## Supplementary Material

zraa038_Supplementary_Data

## Notes

Presented in part to the 25th Annual Conference of the International Society for Quality of Life Research which was held at Dublin, Ireland in October 2018. The meeting abstract was published in Quality of Life Research, Volume 27, Pages S76-S76.
