# Supplementary material for: Psychological distress and health-related quality of life up to 2 years after oesophageal cancer surgery: nationwide population-based study
Source: BJS Open. 2021 Jan 9;5(1):zraa038. doi: 10.1093/bjsopen/zraa038 (PMC7893460; doi:10.1093/bjsopen/zraa038)
Supplement: zraa038_Supplementary_Data [file zraa038_supplementary_data.doc]

**Table S1 Characteristics of patients who had surgical treatment of oesophageal cancer with and without psychological distress at 1, 1.5 and 2 years after surgery**

|  | 1 year | | 1.5 years | | 2 years | |
| --- | --- | --- | --- | --- | --- | --- |
|  | Without psychological distress  (n = 161) | With psychological distress  (n = 19) | Without psychological distress  (n = 130) | With psychological distress  (n = 27) | Without psychological distress  (n = 116) | With psychological distress  (n = 35) |
| **Age at surgery** |  |  |  |  |  |  |
| <60 | 33 (21) | 7 (37) | 22 (17) | 9 (33) | 18 (16) | 10 (29) |
| 60-74 | 102 (63) | 9 (47) | 85 (65) | 15 (56) | 81 (70) | 18 (51) |
| ≥75 | 26 (16) | 3 (16) | 23 (18) | 3 (11) | 17 (15) | 7 (20) |
| **Sex** |  |  |  |  |  |  |
| Female | 21 (13) | 5 (26) | 15 (12) | 7 (26) | 16 (14) | 6 (17) |
| Male | 140 (87) | 14 (74) | 115 (88) | 20 (74) | 100 (86) | 29 (83) |
| **Cohabitation status** |  |  |  |  |  |  |
| Non-cohabitating | 38 (24) | 4 (21) | 28 (22) | 7 (26) | 26 (22) | 7 (20) |
| Cohabitating | 123 (76) | 15 (79) | 102 (78) | 20 (74) | 90 (78) | 28 (80) |
| **Education level** |  |  |  |  |  |  |
| Nine-year compulsory school | 39 (24) | 5 (26) | 33 (25) | 7 (26) | 32 (28) | 7 (20) |
| Upper secondary school | 72 (45) | 9 (47) | 57 (44) | 13 (48) | 47 (41) | 19 (54) |
| Higher education | 50 (31) | 5 (26) | 40 (31) | 7 (26) | 37 (32) | 9 (26) |
| **Charlson Comorbidity Index** | |  |  |  |  |  |
| 0 | 79 (49) | 8 (42) | 64 (49) | 12 (44) | 54 (47) | 18 (51) |
| 1 | 47 (29) | 8 (42) | 37 (28) | 11 (41) | 37 (32) | 12 (34) |
| ≥2 | 35 (22) | 3 (16) | 29 (22) | 4 (15) | 25 (22) | 5 (14) |
| **Neoadjuvant therapy** |  |  |  |  |  |  |
| No | 28 (17) | 5 (26) | 25 (19) | 5 (19) | 22 (19) | 7 (20) |
| Yes | 133 (83) | 14 (74) | 105 (81) | 22 (81) | 94 (81) | 28 (80) |
| **Tumour histology** |  |  |  |  |  |  |
| Adenocarcinoma | 137 (85) | 14 (74) | 112 (86) | 21 (78) | 99 (85) | 26 (74) |
| Squamous cell carcinoma | 24 (15) | 5 (26) | 18 (14) | 6 (22) | 17 (15) | 9 (26) |
| **Surgical approach** |  |  |  |  |  |  |
| Total minimally invasive oesophagectomy | 46 (29) | 4 (21) | 34 (26) | 9 (33) | 30 (26) | 9 (26) |
| Hybrid minimally invasive oesophagectomy | 50 (31) | 9 (47) | 43 (33) | 9 (33) | 41 (35) | 12 (34) |
| Open oesophagectomy | 65 (40) | 6 (32) | 53 (41) | 9 (33) | 45 (39) | 14 (40) |
| **Tumour stage** |  |  |  |  |  |  |
| I | 59 (37) | 8 (42) | 51 (39) | 9 (33) | 49 (42) | 13 (37) |
| II | 54 (34) | 5 (26) | 41 (32) | 10 (37) | 35 (30) | 12 (34) |
| III-IV | 48 (30) | 6 (32) | 38 (29) | 8 (30) | 32 (28) | 10 (29) |
| **Postoperative complications (****Clavien–Dindo grade)** | | |  |  |  |  |
| None | 60 (37) | 5 (26) | 50 (38) | 8 (30) | 47 (41) | 11 (31) |
| I–II | 44 (27) | 6 (32) | 39 (30) | 4 (15) | 36 (31) | 9 (26) |
| III–IV | 57 (35) | 8 (42) | 41 (32) | 15 (56) | 33 (28) | 15 (43) |

**Note.** All values are number (%) and the percentage is rounded up, which in some cases gives a sum not equaling to 100%. No statistically significant difference was found between patients with and without psychological distress at the three time points.

**Table S2 Comparison of characteristics of patients who had surgical treatment of oesophageal cancer between those who did or did not answer the 1.5- or 2-year follow-up questionnaire**

|  | 1.5 years | |  | 2 years | |
| --- | --- | --- | --- | --- | --- |
|  | Answered the questionnaire  (n = 157) | Not answered the questionnaire  (n = 23) |  | Answered the questionnaire  (n = 151) | Not answered the questionnaire  (n = 29) |
| **Age at surgery** |  |  |  |  |  |
| <60 | 31 (20) | 9 (39) |  | 28 (19) | 12 (41) |
| 60-74 | 100 (64) | 11 (48) |  | 99 (66) | 12 (41) |
| ≥75 | 26 (17) | 3 (13) |  | 24 (16) | 5 (17) |
| **Sex** |  |  |  |  |  |
| Female | 22 (14) | 4 (17) |  | 22 (15) | 4 (14) |
| Male | 135 (86) | 19 (83) |  | 129 (85) | 25 (86) |
| **Cohabitation status** |  |  |  |  |  |
| Non-cohabitating | 35 (22) | 7 (30) |  | 33 (22) | 9 (31) |
| Cohabitating | 122 (78) | 16 (70) |  | 118 (78) | 20 (69) |
| **Education level** |  |  |  |  |  |
| Nine-year compulsory school | 40 (25) | 4 (17) |  | 39 (26) | 5 (17) |
| Upper secondary school | 70 (45) | 11 (48) |  | 66 (44) | 15 (52) |
| Higher education | 47 (30) | 8 (35) |  | 46 (30) | 9 (31) |
| **Charlson Comorbidity Index** | |  |  |  |  |
| 0 | 76 (48) | 11 (48) |  | 72 (48) | 15 (52) |
| 1 | 48 (31) | 7 (30) |  | 49 (32) | 6 (21) |
| ≥2 | 33 (21) | 5 (22) |  | 30 (20) | 8 (28) |
| **Neoadjuvant therapy** |  |  |  |  |  |
| No | 30 (19) | 3 (13) |  | 29 (19) | 4 (14) |
| Yes | 127 (81) | 20 (87) |  | 122 (81) | 25 (86) |
| **Tumour histology** |  |  |  |  |  |
| Adenocarcinoma | 133 (85) | 18 (78) |  | 125 (83) | 26 (90) |
| Squamous cell carcinoma | 24 (15) | 5 (22) |  | 26 (17) | 3 (10) |
| **Surgical approach** |  |  |  |  |  |
| Total minimally invasive oesophagectomy | 43 (27) | 7 (30) |  | 39 (26) | 11 (38) |
| Hybrid minimally invasive oesophagectomy | 52 (33) | 7 (30) |  | 53 (35) | 6 (21) |
| Open oesophagectomy | 62 (39) | 9 (39) |  | 59 (39) | 12 (41) |
| **Tumour stage** |  |  |  |  |  |
| I | 60 (38) | 7 (30) |  | 62 (41) | 5 (17) |
| II | 51 (32) | 8 (35) |  | 47 (31) | 12 (41) |
| III-IV | 46 (29) | 8 (35) |  | 42 (28) | 12 (41) |
| **Postoperative complications (Clavien–Dindo grade)** | | |  |  |  |
| None | 58 (37) | 7 (30) |  | 58 (38) | 7 (24) |
| I–II | 43 (27) | 7 (30) |  | 45 (30) | 5 (17) |
| III–IV | 56 (36) | 9 (39) |  | 48 (32) | 17 (59) |
| **Psychological distress at 1 year post-surgery** | | | | | |
| No | 140 (89) | 21 (91) |  | 134 (89) | 27 (93) |
| Yes | 17 (11) | 2 (9) |  | 17 (11) | 2 (7) |

**Note.** All values are number (%) and the percentage is rounded up, which in some cases gives a sum not equaling to 100%.

**Table S3 Adjusted mean score difference (MD) with 95% confidence intervals (CIs) in HRQL aspects between patients who had surgical treatment of oesophageal cancer with and without psychological distress over the three assessment time points: 1, 1.5 and 2 years after surgery (results are from random-effects models**, without significant time interaction)

|  | **Adjusted MD (95% CI; Random effects model)** |
| --- | --- |
| **EORTC QLQ-C30** | |
| Emotional function | **-28 (-32, -24)** |
| Cognitive function | **-14 (-18, -10)** |
| Social function | **-25 (-30, -19)** |
| Dyspnoea | **15 (8, 22)** |
| Fatigue | **17 (12, 22)** |
| Nausea/Vomiting | **8 (3, 13)** |
| Pain | **16 (10, 21)** |
| Insomnia | **22 (15, 29)** |
| Diarrhoea | **9 (2, 15)** |
| Financial difficulty | **12 (7, 16)** |
| Summary score | **-16 (-18, -13)** |
| Physical function | -10 (-14, -6) |
| Role function | -17 (-23, -12) |
| Constipation | 10 (5, 15) |
| **EORTC QLQ-OG25** | |
| Anxiety | **33 (27, 39)** |
| Eating | **18 (13, 23)** |
| Eating with others | **15 (10, 21)** |
| Weight loss | **17 (10, 23)** |
| Odynophagia | **13 (9, 18)** |
| Dry mouth | **14 (8, 21)** |
| Trouble swallowing saliva | **10 (6, 14)** |
| Choked when swallowing | **13 (8, 19)** |
| Dysphagia | 7 (3, 10) |
| Reflux | 6 (-1, 12) |
| Pain and discomfort | 8 (3, 14) |
| Trouble with coughing | 9 (2, 16) |
| Trouble talking | 9 (4, 14) |

**Note.** Values in bold have clinical relevance and statistical significance; MD rounded up to the nearest integer. EORTC QLQ-C30, European Organisation for Research and Treatment of Cancer Quality of Life Questionnaire - Core 30; EORTC QLQ-OG25, European Organisation for Research and Treatment of Cancer Quality of Life Questionnaire - Oesophago-Gastric module 25.

**Table S4 Adjusted mean score difference (MD) with 95% confidence intervals (CIs) in HRQL aspects between patients who had surgical treatment of oesophageal cancer with and without psychological distress** **at 1, 1.5 and 2 years after surgery (results are from random effects models, with significant time interaction)**

|  | **Adjusted MD (95% CI; Random effects model)** | | |
| --- | --- | --- | --- |
|  | **1 year** | **1.5 years** | **2 years** |
| **EORTC QLQ-C30** | | | |
| Global quality of life | **-15 (-22, -7)** | **-21 (-28, -15)** | **-27 (-34, -20)** |
| Appetite loss | 14 (4, 23) | 13 (4, 21) | **31 (23, 40)** |
| **EORTC QLQ-OG25** | | | |
| Trouble with taste | 6 (-3, 16) | **21 (13, 29)** | 9 (1, 18) |
| Body image | **36 (27, 44)** | **29 (22, 37)** | **18 (10, 25)** |

**Note.** Values in bold have clinical relevance and statistical significance; MD rounded up to the nearest integer. EORTC QLQ-C30, European Organisation for Research and Treatment of Cancer Quality of Life Questionnaire - Core 30; EORTC QLQ-OG25, European Organisation for Research and Treatment of Cancer Quality of Life Questionnaire - Oesophago-Gastric module 25.

**Table S5** **Estimated marginal means of health related quality of life (HRQL) at 1 year after surgery and adjusted mean score difference for HRQL between 1, 1.5 and 2 years after surgery among patients who had surgical treatment of oesophageal cancer (results are from fixed-effect models)**

|  | **Patients without anxiety/depression** | | | **Patients with anxiety/depression** | | |
| --- | --- | --- | --- | --- | --- | --- |
|  | **Mean score** | **Mean score difference** | | **Mean score** | **Mean score difference** | |
|  | **1 year** | **1.5 vs. 1 year** | **2 vs. 1 year** | **1 year** | **1.5 vs. 1 year** | **2 vs. 1 year** |
| **EORTC QLQ-C30** | | | | | | |
| Global quality of life | 71 (69, 73) | 0 (-3, 3) | 0 (-3, 4) | 66 (57, 74) | -8 (-17, +0) | **-14 (-23, -6)** |
| Cognitive function | 87(85, 88) | -1 (-4, 1) | 0 (-3, 3) | 79 (73, 86) | 3 (-3, 9) | -4 (-10, 3) |
| Appetite loss | 16 (13, 18) | 0 (-4, 4) | 0 (-4, 4) | 21 (11, 32) | 1 (-10, 11) | **21 (10, 31)** |
| Summary score | 83 (82, 85) | -4 (-6, -2) | -4 (-6, -2) | 75 (70, 80) | -4 (-8, 1) | -9 (-14, -5) |
| **EORTC OG25** | | | | | |  |
| Trouble with taste | 16 (13, 18) | 0 (-3, 4) | 3 (-1, 7) | 12 (1, 22) | **18 (8, 28)** | **11 (1, 22)** |
| Body image | 10 (7, 12) | 2 (-2, 5) | 4 (1, 8) | 37 (28, 47) | -3 (-13, 6) | **-10 (-20, -1)** |

**Note.** Values in bold have clinical relevance and statistical significance; all values rounded up to the nearest integer; 95% confidence intervals are presented in parentheses. vs.: versus; EORTC QLQ-C30, European Organisation for Research and Treatment of Cancer Quality of Life Questionnaire - Core 30; EORTC QLQ-OG25, European Organisation for Research and Treatment of Cancer Quality of Life Questionnaire - Oesophago-Gastric module 25.
